# Management of leiomyosarcomas of the spermatic cord: the role of reconstructive surgery

**DOI:** 10.1186/1477-7819-3-23

**Published:** 2005-04-28

**Authors:** Stuart Enoch, Simon M Wharton, Douglas S Murray

**Affiliations:** 1 West Midlands Regional Centre for Plastic and Reconstructive Surgery, Selly Oak Hospital, University Hospital of Birmingham, – B29 6JD, UK; 2 Wound Healing Research Unit, University Department of Surgery, University of Cardiff/University Hospital of Wales, Cardiff, – CF14 4UJ, UK

## Abstract

**Background:**

Leiomyosarcomas (LMS) of the spermatic cord are extremely rare. Radical inguinal orchiectomy and high ligation of the cord is the standard primary surgical procedure. The extent of surrounding soft tissue excision required and the precise role of adjuvant radiotherapy, however, remains unclear. In addition, recurrence is a commonly encountered problem which might necessitate further radical excision of adjacent soft tissues.

**Methods:**

This article reviews the pathophysiology of spermatic cord leiomyosarcomas (LMS), and discusses the various reconstructive surgical options available to repair the inguinal region and the lower anterior abdominal wall after excision of the tumour and the adjacent soft tissues.

**Results:**

There is paucity of literature on LMS of spermatic cord. The majority of paratesticular neoplasms are of mesenchymal origin and up to 30% of these are malignant. In adults, approximately 10% of spermatic cord sarcomas are LMS. Approximately 50% of these tumours recur loco-regionally following definitive surgery; however, the incidence decreases if resection is followed by adjuvant radiotherapy.

**Conclusion:**

It is therefore important to achieve negative histological margins during the primary surgical procedure, even if adjuvant radiotherapy is instituted. If extensive resection is required, either during the primary procedure or following recurrence, reconstructive surgery may become necessary. This article reviews the pathophysiology of spermatic cord LMS, the reasons for recurrence, and discusses the management options including the role of reconstructive surgery.

## Introduction

Tumours of the spermatic cord and paratesticular tissue are rare [1,2], and as such, their true incidence has never been established. Radical inguinal orchiectomy and high ligation of the cord is the standard primary surgical procedure. However, the extent of surrounding soft tissue excision required, including margins, and the role of adjuvant radiotherapy (RT), remains controversial. The paucity of literature in this area often makes treatment decisions difficult; prospective trials are precluded by the rarity of this tumour and no series is sufficiently large to accurately evaluate the most appropriate treatment option.

Recurrence is a commonly encountered problem, which might necessitate further radical excision of adjacent soft tissues. It hence becomes important to achieve negative histological margins during the primary surgery even if adjuvant radiotherapy (RT) is instituted. If extensive areas of adjacent soft tissues are resected, either during the primary procedure or following recurrence, reconstructive surgery may be indicated to repair the defect.

In addition to debating the above issues, this article reviews the pathophysiology of spermatic cord leiomyosarcomas (LMS), and discusses the various reconstructive surgical options available to repair the inguinal region and the lower anterior abdominal wall after excision of the tumour and the adjacent soft tissues.

### Pathophysiology

The majority of paratesticular neoplasms are of mesenchymal origin and up to 30% of these are malignant [3]. In adults, approximately 10% of spermatic cord sarcomas are LMS with a peak incidence in the sixth and seventh decades [4]. LMS most likely originate from the smooth muscle of different areas such as the vas deference canal wall, blood vessels and cremaster muscle, and is thought to arise as a result of malignant degeneration from previously existing leiomyomatous tumours [5].

It has been estimated that approximately 50% of these tumours recur loco-regionally following definitive surgery [6-10], although the incidence decreases if resection is followed by adjuvant RT [8,9]. Pathologic features that convey a higher risk of local recurrence include large tumour size, inguinal location, narrow or positive margins and prior intralesional surgery [11]. Loco-regional relapse may occur in the cord, scrotum, or adjacent pelvis, with or without involvement of the regional lymph nodes [10]. If relapse occurs in the cord or scrotum, they often extend proximally through the internal inguinal ring into the pelvic cavity [12].

The patients may also present with signs and symptoms of distant metastasis, although dissemination may be seen as late as 15 or more years after resection of the primary tumour [13]. The common means of metastasis are by lymphatic spread either to the regional (pelvic) [12] or distant lymph nodes (para-aortic) [14], and by the haematogenous route, usually to the lungs and the liver [9,12]; metastasis to other regions such as the orbit [15], although rare, may also occur.

### Management

Preoperative diagnosis of spermatic cord LMS is difficult and the tumour is usually described as a firm, gradually enlarging, and painless extra-testicular intra-scrotal mass [16]. It may be occasionally accompanied by a hydrocoele [1]. Scrotal ultrasound is a useful initial investigation to identify a sinister mass within the scrotum [17]. If a neoplasm is identified, magnetic resonance imaging is the investigation of choice to characterise and delineate the anatomical extent of the tumour [18]. Other modalities such as fluorodeoxyglucose positron emission tomography (FDG-PET) have been used in the diagnosis of dedifferentiated liposarcoma of the spermatic cord [19]; but it is not established in routine clinical practice. The role, if any, of fine needle aspiration cytology in the preoperative diagnosis of spermatic cord LMS has not been clearly established although they have been used to diagnose other types of spermatic cord sarcomas such as liposarcoma [20] and malignant fibrous histiocytoma [21,22].

#### Surgical excision and margins

Radical inguinal orchiectomy and high ligation of the cord is the standard primary surgical procedure [9,23,24]. The extent of surrounding soft tissue excision and the margins required, however, remains controversial. Simple excision is clearly inadequate for paratesticular sarcomas as repeat wide excision has revealed microscopic residual disease in 27% of completely excised cases [23]. Aggressive surgical strategies are therefore recommended in the management of spermatic cord LMS and a decrease in local recurrence has been observed in patients who underwent re-operative wide excision after a prior incomplete resection [25].

Malignant mesenchymal tumours such as LMS have a "pseudocapsule" [26] and, as such, there may be infiltration of tumour cells into the adjacent tissues; therefore, it might be difficult to precisely estimate the tumour margins. Due to this 'irregular' pattern of tumour growth and the anatomical constraints in the inguinal region, wide circumferential resection margins may be difficult to achieve [1,12]. Nevertheless, due to the tumours' high propensity for local recurrence, if negative histological margins are not achieved during primary surgery, re-excision should be considered mandatory, even if this involves sacrificing some areas of adjacent normal anatomy. Advances in microsurgery have made it possible to reconstruct significant anatomical defects in this region (discussed later).

The role of prophylactic lymph node dissection remains unclear. Although performed in some centres, the true incidence of para-aortic and pelvic nodal metastasis has never been documented [12], and hence there is insufficient evidence at present to suggest that prophylactic para-aortic and pelvic nodal dissection prevents relapse or improves the prognosis of patients with spermatic cord LMS [8]. It, however, has a role in other types of para-testicular sarcomas such as rhabdomyosarcomas, fibrosarcomas, and intermediate or high-grade malignant fibrous histiocytomas [7].

#### Surgically relevant anatomy

**Figure 1:**
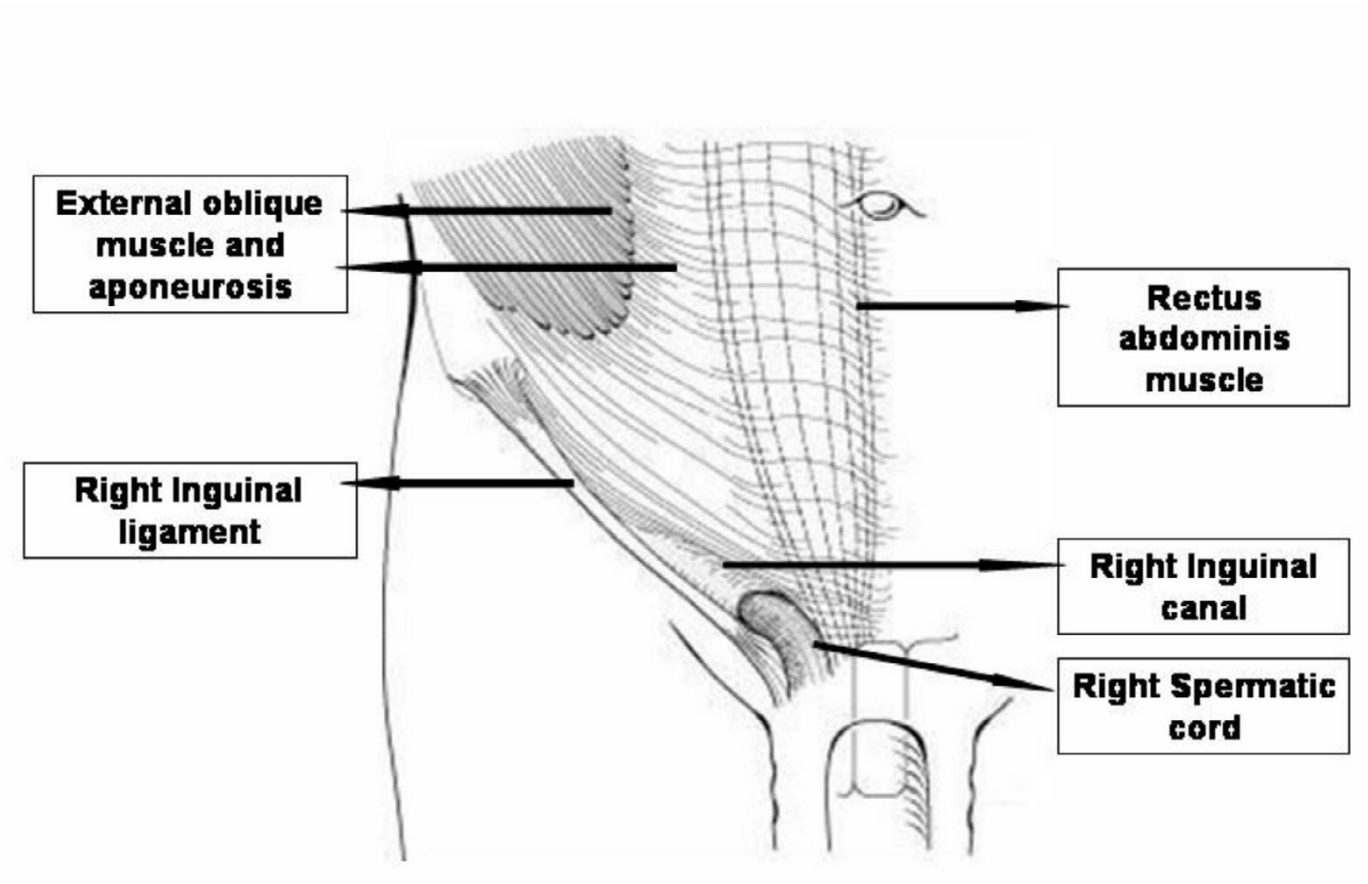
Layers of the anterior abdominal wall

**Figure 2:**
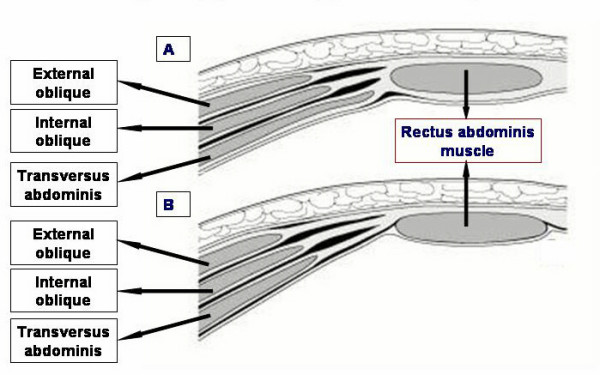
Formation of the rectus sheath in horizontal section, above [A] and below [B] the arcuate line

The inguinal region lies at the junction of the lower abdomen and the anterior thigh. The lower abdominal wall below skin and subcutaneous tissue is composed of three layers of muscle (external oblique, internal oblique and transversus abdominis) and their aponeurosis anterolaterally, and vertical rectus muscle covered in an aponeurotic sleeve medially (Figure 1). The rectus sheath differs in composition above and below the arcuate line, which lies one-third of the way from the umbilicus to the symphysis pubis (Figure 2).

The inguinal canal allows the passage of the spermatic cord or round ligament (in females) without vascular compromise and without obstructing ductus deferens in the male. Below the inguinal ligament lies the femoral triangle. The boundaries of the femoral triangle are the inguinal ligament superiorly, the medial border of sartorius laterally, and the medial border of adductor longus medially. The femoral triangle contains the femoral vessels and nerve, sapheno-femoral junction and the deep inguinal lymph nodes.

#### Reconstruction of the inguinal region and the anterior lower abdominal wall after wide surgical resection

**Figure 3:**
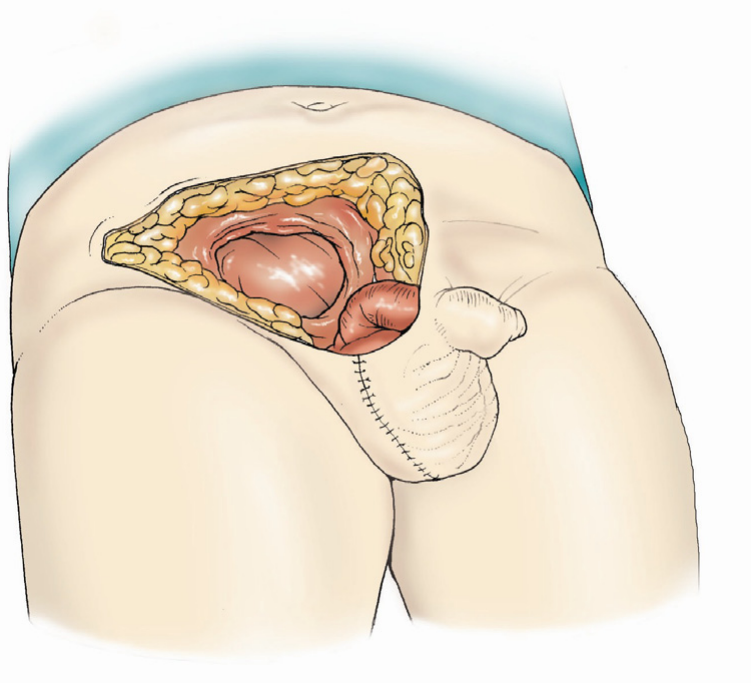
Defect created after removal of the tumour along with lower part of the abdominal wall including the inguinal canal, part of rectus sheath and external oblique aponeurosis, subcutaneous tissue and skin

**Figure 4:**
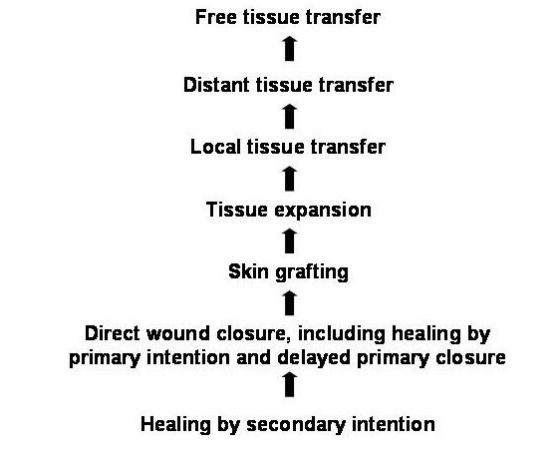
Reconstructive ladder in wound closure

After wide excision of soft tissues, it is important to repair the defect in the inguinal region and the lower anterior abdominal wall (Figure 3). The structures that may require covering include the spermatic cord, femoral neurovascular bundle, abdominal wall muscles and sometimes-exposed bone. If the surgical resection involves removal of abdominal wall muscles, then reconstruction using mesh in addition to tissue coverage may be necessary. The principle of soft tissue reconstruction is based on the reconstructive ladder (Figure 4). Various reconstructive surgical options are available in the plastic surgeon's armoury to repair defects in this region (Table 1).

**Table 1:** reconstructive surgical options to cover defects in the inguinal region and the anterior lower abdominal wall

**Coverage type**	**Name of flap**
Skin and fat flap	• Deep inferior epigastric perforator (DIEP) flap
Fascio-cutaneous flaps	• Antero – lateral thigh flap
	• Groin flap
Muscle/musculo-cutaneous flaps	• Rectus abdominis flap (TRAM/VRAM)
	• Tensor fasciae latae flap
	• Gracilis flap
	• Rectus femoris flap
	• Vastus lateralis flap
Free flap	• Latissimus dorsi flap
Others	• Sartorius muscle switch
	• Omental flap

**Figure 5:**
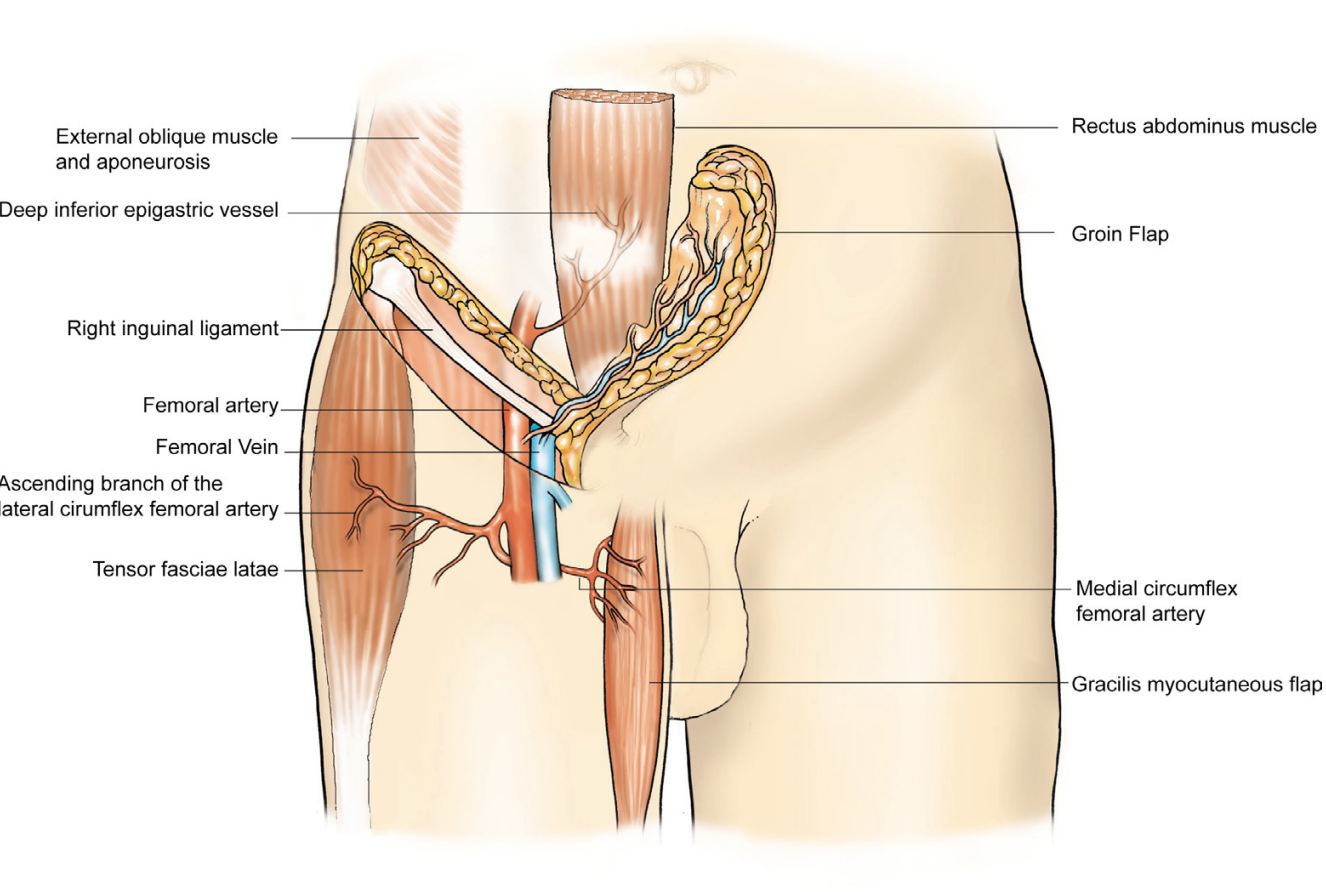
Some reconstructive options (tensor fasciae latae flap, gracilis flap, rectus abdominus flap, groin flap) available to repair defects in the inguinal region and the lower anterior abdominal wall

Local flaps to cover the inguinal region and the lower anterior abdominal wall include the muscle or musculo-cutaneous flaps such as rectus abdominis (transverse rectus abdominis muscle (TRAM) or vertical rectus abdominis muscle (VRAM) flaps, gracillis flap, tensor fasciae latae flap (Figure 5), rectus femoris flap and vastus lateralis flap. The tensor fasciae latae (TFL) flap is a frequently employed flap because it is very resilient and has a reliable vascular pedicle from the lateral femoral circumflex branch of the profunda femoris artery [27] (Figure 5). In addition, the division of TFL from its normal anatomical insertion does not have a significant effect on the function of the limb; for this reason, the rectus femoris and vastus lateralis flaps are not the first choice since their loss may lead to some limitation of movements at the hip or knee joints, and thus functional loss.

If the resection is not deep (no muscle loss), fascio-cutaneous flaps such as the groin flap and the antero-lateral thigh flap, or an abdominal wall skin and fat flap based on perforating vessels – deep inferior epigastric perforator (DIEP) flap – could be used. The use of a groin flap, however, may be limited in situations where the vessels, which originate in the femoral triangle, may be ligated as part of the resection. If femoral dissection is required leading to soft tissue loss, a sartorius muscle switch or an omental flap [28] could be used to cover exposed vessels in the femoral triangle.

In the event of no local flap options being available (very rare), a free flap may be used to achieve cover. This involves raising a distant flap, usually the latissimus dorsi muscle, disconnecting the blood supply and transferring the tissue to the groin and anastomosing the thoraco-dorsal vessels to recipient vessels in the inguinal region.

The use of well vascularised tissue to cover exposed femoral vessels is important as it minimises local vascular complications when adjuvant RT is instituted to the groin region.

The various reconstructive options discussed above are useful in different clinical (or operative) scenarios. With adequate planning and proper choice, satisfactory short- and long-term results can be obtained with all the above reconstructive procedures. The choice of one particular flap for reconstruction depends on various factors: (i) the anatomical location of the cover required; (ii) the extent and type of soft tissue loss (e.g., skin, muscle, etc.); (iii) tumour type – primary or recurrent; (iv) the ease of donor site coverage; (v) flap availability; and (vi) the experience of the surgeon in the usage of a particular flap. In primary tumours, the skin might not be involved and hence a muscle flap might be adequate. However, in recurrence, the overlying skin might be damaged due to RT. This may necessitate excision of the skin (along with underlying tissues) and coverage using a musculo-cutaneous or a fascio-cutanoeus flap (alternatively a muscle flap with split skin grafting can be used).

#### Radiotherapy and chemotherapy

Adjuvant RT has become an established method in the management of LMS of the spermatic cord [8,9,12,13]. Some authors recommend adjuvant RT only for high-grade LMS or to those patients believed to be at a high risk of local recurrence. However, due to the tumour's high propensity (approximately 50%) [8,9] of local recurrence following surgery alone [6,12], there is increasing consensus that LMS of all grades and histology should receive adjuvant RT [12]. Several studies have reported better results with combined modality treatment and the recurrence could be reduced to 10 – 20% by using adjuvant RT [29]. It needs to be emphasised at this point that adjuvant RT should supplement rather than substitute radical surgical excision and should be instituted only after complete clearance of the tumour is achieved surgically. The field for RT should include the inguinal canal, ipsilateral pelvic tissue [12], and the scrotum [8]. The role of pre-operative RT has not been clearly established, and, as such, there is no evidence to suggest that radiotherapy before surgical excision reduces the rate of recurrence or improves the overall prognosis.

There are no controlled studies at present that specifically addresses the role of adjuvant systemic chemotherapy in adult spermatic cord LMS [11], although they have a well-defined role in childhood rhabdomyosarcomas [30]. Adjuvant chemotherapy is currently not used routinely in the management of spermatic cord LMS though it has been suggested that it might have a role in abrogating the haematogenous metastatic potential in high grade sarcomas [12] and in patients with metastatic disease.

### Prognosis

The prognosis of patients with LMS is highly variable. Kyle (1966) [31] suggested a probable five-year survival of 10–15% but more recently a five-year survival of 50–80% has been reported [9], possibly reflecting the advances in diagnosis and management of these tumours. The wide range in the five-year survival rate might be due to the variations in tumour stage and grade at the time of diagnosis as well as the diversity of therapies involved.

## Conclusion

In the management of spermatic cord LMS, complete excision of the tumour with radical inguinal orchiectomy and high ligation of the cord should be the primary surgical procedure. Due to the high propensity for local recurrence, if the margins are not clear, re-excision should be considered in all cases. In addition, based on current evidence, the authors recommend that adjuvant RT should be instituted for all grades and types of spermatic cord LMS. There is insufficient evidence to advocate prophylactic nodal treatment in the management of these tumours.

## Competing interests

The author(s) declare that they have no competing interests

## Authors' contributions

**SE **– Conception and design, literature review with references, drafting the article and revising, preparation of images

**SMW **– Drafting the article and revising, preparation of images

**DSM **– Specialist plastic surgical input, supervision, and review, proof-reading and revising the manuscript

All authors read and approved the final manuscript for publication
